# Effect of Applying Leakage Correction on rCBV Measurement Derived From DSC-MRI in Enhancing and Nonenhancing Glioma

**DOI:** 10.3389/fonc.2021.648528

**Published:** 2021-03-23

**Authors:** Fatemeh Arzanforoosh, Paula L. Croal, Karin A. van Garderen, Marion Smits, Michael A. Chappell, Esther A. H. Warnert

**Affiliations:** ^1^ Department of Radiology and Nuclear Medicine, Erasmus MC, Rotterdam, Netherlands; ^2^ Radiological Sciences, Mental Health and Clinical Neurosciences, School of Medicine, University of Nottingham, Nottingham, United Kingdom; ^3^ Sir Peter Mansfield Imaging Centre, School of Medicine, University of Nottingham, Nottingham, United Kingdom; ^4^ NIHR Nottingham Biomedical Research Centre, Queen’s Medical Centre, University of Nottingham, Nottingham, United Kingdom

**Keywords:** dynamic susceptibility contrast (DSC), relative cerebral blood volume (rCBV), unidirectional leakage correction, bidirectional leakage correction, glioma

## Abstract

**Purpose:**

Relative cerebral blood volume (rCBV) is the most widely used parameter derived from DSC perfusion MR imaging for predicting brain tumor aggressiveness. However, accurate rCBV estimation is challenging in enhancing glioma, because of contrast agent extravasation through a disrupted blood-brain barrier (BBB), and even for nonenhancing glioma with an intact BBB, due to an elevated steady-state contrast agent concentration in the vasculature after first passage. In this study a thorough investigation of the effects of two different leakage correction algorithms on rCBV estimation for enhancing and nonenhancing tumors was conducted.

**Methods:**

Two datasets were used retrospectively in this study: 1. A publicly available TCIA dataset (49 patients with 35 enhancing and 14 nonenhancing glioma); 2. A dataset acquired clinically at Erasmus MC (EMC, Rotterdam, NL) (47 patients with 20 enhancing and 27 nonenhancing glial brain lesions). The leakage correction algorithms investigated in this study were: a unidirectional model-based algorithm with flux of contrast agent from the intra- to the extravascular extracellular space (EES); and a bidirectional model-based algorithm additionally including flow from EES to the intravascular space.

**Results:**

In enhancing glioma, the estimated average contrast-enhanced tumor rCBV significantly (Bonferroni corrected Wilcoxon Signed Rank Test, p < 0.05) decreased across the patients when applying unidirectional and bidirectional correction: 4.00 ± 2.11 (uncorrected), 3.19 ± 1.65 (unidirectional), and 2.91 ± 1.55 (bidirectional) in TCIA dataset and 2.51 ± 1.3 (uncorrected), 1.72 ± 0.84 (unidirectional), and 1.59 ± 0.9 (bidirectional) in EMC dataset. In nonenhancing glioma, a significant but smaller difference in observed rCBV was found after application of both correction methods used in this study: 1.42 ± 0.60 (uncorrected), 1.28 ± 0.46 (unidirectional), and 1.24 ± 0.37 (bidirectional) in TCIA dataset and 0.91 ± 0.49 (uncorrected), 0.77 ± 0.37 (unidirectional), and 0.67 ± 0.34 (bidirectional) in EMC dataset.

**Conclusion:**

Both leakage correction algorithms were found to change rCBV estimation with BBB disruption in enhancing glioma, and to a lesser degree in nonenhancing glioma. Stronger effects were found for bidirectional leakage correction than for unidirectional leakage correction.

## Introduction

Dynamic susceptibility contrast (DSC)-MRI is a technique that uses rapid measurements of MRI signal change following the injection of a gadolinium-based contrast agent (GBCA) (1). Perfusion parameters derived from DSC-MRI are increasingly utilized as image-based biomarkers for management of patients with brain cancer. Of particular interest is relative cerebral blood volume (rCBV). It is the most widely used parameter derived from DSC-MRI for predicting brain tumor aggressiveness (2, 3). rCBV also has the potential to predict overall survival of brain tumor patients (4) and can be used in brain tumor monitoring, where it may have value in early detection of local recurrence or malignant transformation, and can aid in differentiation of posttreatment changes from tumor recurrence (5).

A particular challenge in using DSC-MRI for the determination of rCBV in brain tumors is that the presence of a leaky blood-brain barrier (BBB) may confound measurements (6). GBCA leads to shortening of effective transverse relaxation time ${\text{T}}_{2}^{*}$ and shortening of longitudinal relaxation time T_1_. In ${\text{T}}_{2}^{*}$-weighted DSC-MRI acquisition, the shortening of ${\text{T}}_{2}^{*}$ results in signal loss induced by the passage of the paramagnetic contrast agent. This forms the basis of rCBV estimation. In lesions with a disrupted BBB, GBCA leaks into the extravascular extracellular space (EES), reducing both ${\text{T}}_{2}^{*}$ time and T_1_ time even further. In DSC-based perfusion quantification, this phenomenon violates underlying assumptions and thus could lead either to an under- or overestimation of rCBV, depending on the dominant leakage effect (7). A disrupted BBB is present in enhancing glioma, defined as a glial tumor in which a signal increase is clearly seen on T_1_-weighted imaging after injection of a GBCA.

Various strategies have been proposed to address the GBCA leakage issue in DSC-MRI; however, no universally accepted method currently exists (8). Many of these techniques concentrate on the reduction of T_1_ effects, such as application of a preload bolus injection of contrast agent or optimizing acquisition parameters such as echo time, repetition time and flip angle (9, 10). Using a preload prior to the bolus injection for the DSC acquisition is done to saturate the EES and thereby diminish the T_1_ induced signal intensity increase during the subsequent DSC-MRI GBCA administration (11). A number of post-processing methods have also been proposed to correct both ${\text{T}}_{2}^{*}-$ and T_1_-related leakage effects, either by themselves or in addition to advanced acquisition methods (12–15). Among first published post processing methods for addressing GBCA leakage in glioma was the model-based approach by Boxerman–Schmainda–Weisskoff (12). Known as the BSW leakage correction method, it aims to correct both ${\text{T}}_{2}^{*}-$ and T_1_-related leakage effects by modeling the temporal curves of transverse relaxation rate changes in tumor voxels using two terms: one derived from the average relaxivity in nonenhancing tissues, where there is no contrast agent leakage, and the other term that models contrast agent flux from the intravascular space to the EES, with the assumption of no contrast agent back flux.

The BSW method has been widely used and implemented by several commercial software vendors (16). It has been shown that rCBV measurements resulting from a DSC acquisition acquired after a preload and with using the BSW method agree well with histology in spatially correlated tissue biopsies in patients diagnosed with high grade glioma (17, 18). Despite this promising result, limitations of the BSW approach prompted researchers to look for modifications to this leakage correction method. Leigh et al. (13) introduced an arrival time correction to this model, in order to solve mismatch of mean transit time between normal and malignant tissue. Bjørnerud et al. (14) estimated leakage from the residue function, obtained *via* singular value decomposition (SVD), to distinguish between T_1_ and ${\text{T}}_{2}^{*}$ dominant extravasation effects. Recently, the BSW model was extended with bidirectional contrast agent exchange, additionally including flow from EES to the intravascular space (15, 19). Considering that contrast agent exchange is in principle bidirectional, this modification could potentially improve the accuracy of rCBV estimates.

In light of the rising use of rCBV and other DSC-based biomarkers and with current recommendations for acquiring DSC-MRI data including a preload bolus (20), there is an increasing demand for guidance on accurate leakage correction in clinical settings. Moreover, the necessity of using model-based postprocessing leakage correction has been highlighted for high-grade gliomas (20), where typically a contrast-enhancing lesion is seen in T_1_-weighted postcontrast images. However, to the best of our knowledge, the effect of using leakage correction algorithm on nonenhancing glioma, when there is no visually detectable contrast enhancing lesion in T_1_-weighted postcontrast, has not been investigated. Fully understanding the effects of applying leakage correction for rCBV estimation can help the radiologists and technicians using commercial or free software for analyzing DSC-MRI data decide if they need to “tick the box” of leakage correction for both enhancing and nonenhancing tumor. Therefore, this study focuses on a thorough investigation of the effect of using the well-known BSW leakage correction algorithm (12) as well as its recent modified leakage correction algorithm (19) on rCBV estimation in both enhancing and nonenhancing glioma, using two different datasets acquired with different parameters and different GBCA dosage. In the following, we will refer to the former method as the unidirectional and the latter as the bidirectional leakage correction algorithm.

## Materials and Methods

### The Theory

In DSC-MRI, the dynamic signal drop caused by passage of an intravascular GBCA bolus is assumed to be proportional to the change in concentration of GBCA over time, causing a proportional change in relaxation rate (21), as expressed by equation [1]: 

(1) $$C(t)\;\propto \;\Delta R_{2}^{*}(t)=-(1/T_{E})\times (ln(S(t)/S_{0}))$$

where $\Delta R_{2}^{*}(t)$ is the inverse of the change in ${\text{T}}_{2}^{*}$ relaxation time (relaxivity-time curve), C(t) is the tracer concentration at time t, S(t) is the intensity time-signal, S_0_ is the baseline signal in the voxel prior to the contrast bolus arrival, and T_E_ is the echo time. The uncorrected rCBV is estimated by trapezoidal integration between entrance t_0_ and exit t_1_ time points of the bolus in the relaxivity-time curve:

(2) $$rCBV=\int_{t_{0}}^{t_{1}}\Delta R_{2}^{*}(t)\;dt$$

In the unidirectional leakage correction algorithm, the measured relaxivity change $\Delta R_{2}^{*}(t)$ for each voxel can be modeled as a linear combination of the whole-brain average relaxivity-time curve $\bar{\Delta R_{2}^{*}}(t)$ in nonenhancing voxels and its time integral:

(3) $$\Delta R_{2}^{*}(t)\approx K_{1}\;\bar{\Delta R_{2}^{*}}(t)-K_{2}\;\int_{0}^{t}\bar{\Delta R_{2}^{*}}(t')\;dt'$$

where K_1_ (sec^-1^) is a susceptibility scaling factor, K_2_ (sec^-1^) is a permeability related parameter for intra- to extravascular contrast flux and both are estimated by a linear least square fit of the measured $\Delta R_{2}^{*}(t)$ to equation [3]. Thus, the unidirectional corrected relaxivity-time curve $\Delta R_{2\text{ unidir}}^{*}$ and rCBV_unidir_ can be calculated for each voxel: 

(4) $$\Delta R_{2\;unidir}^{*}(t)=\Delta R_{2}^{*}(t)+K_{2}\;\int_{0}^{t}\bar{\Delta R_{2}^{*}}(t')\;dt'$$

(5) $$rCBV_{unidir}=\int_{t_{0}}^{t_{1}}\Delta R_{2\;unidir}^{*}(t)\;dt$$

In the bidirectional leakage correction algorithm, the assumption is that contrast agent flows back and forth between the intravascular and extravascular compartment. This is implemented by adding an extra term to equation [3] where $\Delta R_{2}^{*}(t)$ can be modeled as follows:

(6) $$\Delta R_{2}^{*}(t)\approx K_{1}\;\bar{\Delta R_{2}^{*}}(t)-K_{2}\;\int_{0}^{t}\bar{\Delta R_{2}^{*}}(t')\;e^{-K_{ep}(t-t')}dt'$$

where K_ep_ is the transfer coefficient for extra- to intravascular contrast flux and substituting K_ep_=0 yields the unidirectional leakage correction equation [3]. After applying least square fitting, and obtaining K_1_, K_2_ and K_ep_ the bidirectional corrected relaxivity-time curve $\Delta R_{2\text{ bidir}}^{*}$ and rCBV_bidir_ can be calculated for each voxel:

(7) $$\Delta R_{2\;bidir}^{*}(t)=\Delta R_{2}^{*}(t)+K_{2}\;\int_{0}^{t}\bar{\Delta R_{2}^{*}}(t')\;e^{-k_{ep}(t-t')}\;dt'$$

(8) $$rCBV_{bidir}=\int_{t_{0}}^{t_{1}}\Delta R_{2\;bidir}^{*}(t)\;dt$$

Note that we did not impose any constraints for fitting K_ep_, K_2_ and K_1_ in any of those methods. This allows K_2_ to be positive or negative in both methods to account for T_1_ and ${\text{T}}_{2}^{*}$ leakage effects.

### Patients and MR Imaging

Two datasets were used retrospectively in this study. The first dataset, “Glioma DSC-MRI Perfusion Data”, is publicly available in The Cancer Imaging Archive (TCIA) (22, 23). This dataset contains 49 patients (51 ± 16 years, 31 male) with coregistered DSC-MRI and post contrast T_1_-weighted SPGR images of nonenhancing (n = 14) and enhancing (n = 35) glioma. These MR images were acquired at 1.5T or 3T on systems from two vendors (GE Healthcare, Waukesha, WI, US; Siemens, Erlangen, DE). All patients had received 0.1 mmol/kg of gadobenate dimeglumine (MultiHance, Bracco Diagnostics, Cranbury, NJ, US) during DSC-MRI acquisition with gradient-echo echo-planar imaging (Repetition Time (T_R_): 1.1/1.25 s, Echo Time (T_E_): 30 ms, Flip Angle (FA): 70/72/80°, 120 dynamics, voxel size: 0.85 × 0.85 × 6.5 mm^3^, 13 slices), preceded by the injection of 0.05 mmol/kg of the same contrast agent as preload bolus, except for one patient who had received 0.01 mmol/kg for preload. A power injector was used for contrast agent injection with antecubital injection and typically set at a rate of 3 ml/s. The parameters for T_1_-weighted postcontrast are not consistent between all patients (T_R_ ranging from 34 ms to 666 ms, T_E_ ranging from 2.3 ms to 21 ms).

The second dataset collected at the Erasmus MC (EMC, Rotterdam, the Netherlands) contained 47 patients (50 ± 10 years, 32 male) with coregistered T_1_-weighted (FSPGR) and DSC-MRI images from patients with confirmed enhancing (n = 20) and nonenhancing (n = 27) glioma. These patients underwent MRI scans at 3T (Discovery MR750, GE, Waukesha, USA) with preload administration of 7.5 ml of gadobutrol 1.0 mmol/ml (Gadovist^®^1.0, Bayer AG, Leverkusen, DE) followed by administration of the same dose of gadobutrol during DSC acquisition with GRE echo-planar imaging (T_R_: 2 s, T_E_:45 ms, FA: 90°, 50 dynamics, voxel size: 2 × 2 × 5 mm^3^, 26 slices). The contrast agent injection was done by power injector with an injection rate of 5 ml/s of the bolus as well as the saline flush (20 ml, following the contrast agent bolus). Additionally, high resolution inversion recovery T_1_-weighted pre- and postcontrast images (T_R_: 2.1 ms, T_E_: 4.6 ms, voxel size: 0.5 × 0.5 × 0.8 mm^3^), structural images of T_2_-weighted (T2W) (T_R_: 0.14 s, T_E_: 8.4 s, voxel size: 0.46 × 0.46 × 5.00 mm^3^) and FLAIR (Fluid-Attenuated Inversion Recovery) (T_R_: 1.7 s, T_E_: 90 ms, voxel size: 0.54 × 0.54 × 0.79 mm^3^) were collected in this dataset. The complete protocol is part of routine clinical imaging and all patients provided informed written consent to have their information stored in an Institutional Review Board Approved Neuro-Oncology database for use in future investigations.

In both datasets separation of enhancing and nonenhancing glioma patients was done by visual inspection of pre- and post-contrast T_1_-weighted imaging, by a certified neuroradiologist with more than 12 years of experience for EMC dataset and a neuroradiologist with more than 20 years of experience for TCIA dataset

### Volume of Interest Delineation

For the TCIA dataset the provided volumes of interest (VOI) were used. These were binary masks of the whole brain, the contrast enhanced part of the tumor mask for enhancing glioma (CET), non-contrast enhancing part of tumor for nonenhancing glioma (NCET) and normal appearing white matter mask (NAWM) (8). These masks had been drawn manually on structural images by an experienced radiologist and all were coregistered to the DSC-MRI dataset.

For the EMC dataset, we used HD-BET for brain extraction of the T_1_-weighted images to generate brain masks (24). FAST (FMRIB’s Automated Segmentation Tool) (25) was used to generate probability maps of white matter, grey matter and cerebrospinal fluid. The NAWM mask was obtained by thresholding and binarizing probability maps of white matter (probability>0.90) in the contralateral part of brain. This binarized map was eroded using **FSL** tools (http://www.fmrib.ox.ac.uk/fsl/) to generate NAWM masks comparable in size with the NAWM masks in TCIA dataset. The generated NAWM mask encompasses on average 50 voxels covering multiple slices and was used for rCBV normalization.

For tumor segmentation, first structural images of T_1_-weighted precontrast, T_2_-weighted and FLAIR were registered to T_1_-weighted postcontrast using the Elastix toolbox (version 2.5) (26). Then, based on these 4 structural images, NCET mask for nonenhancing as well as CET mask for enhancing glioma were delineated using HD-GLIO (27, 28).

### Relative Cerebral Blood Volume Measurements

**Figure 1:**
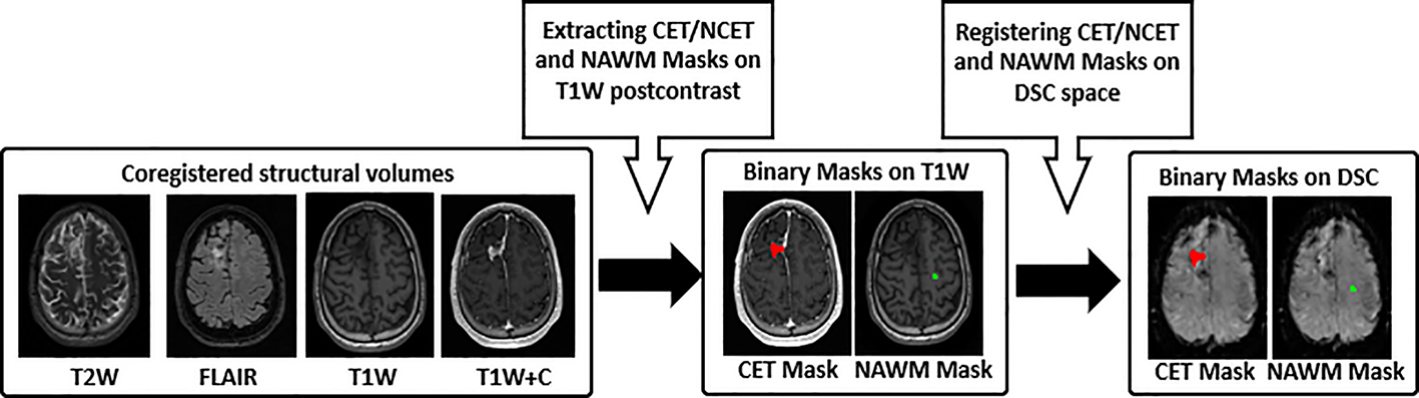
Pre-processing workflow. 1) T_2_-weighted (T2W), FLAIR, T_1_-weighted precontrast (T1W) and postcontrast (T1W+C) images used for CET and NCET, and NAWM segmentation, 2) Binary mask of CET/NCET (red color) and NAWM (green color) mask on the structural data, 3) Registered Binary mask of CET/NCET on the third volume of DSC-MR imaging. CET, Contrast Enhanced Tumor; NCET, Non Contrast Enhanced Tumor; NAWM, Normal Appearing White Matter.

In processing the DSC datasets, two first brain volumes from each individual DSC dataset were removed to make sure that the GRE signal had reached steady state. Then, all masks as well as other volumes of the DSC dataset were rigidly registered to the third volume of DSC-MRI dataset using FLIRT (FMRIB’s Linear Image Registration Tool) (29, 30) (see **Figure 1**).

In-house code developed in Python 3.6 (http://www.python.org) was used for image analysis. To ensure sufficient contrast-to-noise for the time curves, we excluded voxels exhibiting a drop of fewer than 5 standard deviations from the baseline signal from the analysis (31). DSC signal–time curves were converted to relaxivity–time curves using equation [1]. To fit equation [4] and equation [7], relaxivity–time curves of nonenhancing voxels were selected and averaged to produce $\bar{\Delta R_{2}^{*}}(t)$ needed in both leakage correction methods. Those nonenhancing voxels were selected from voxels in the brain mask where the absolute difference between average signal of the tail (final 10 time points) and the baseline (timepoints prior to the contrast bolus arrival) was less than one standard deviation of the baseline signal (12).

Trapezoidal integration between entrance and exit bolus time points of the 3 relaxivity–time curves, uncorrected, unidirectionally and bidirectionally corrected, was then used to obtain rCBV, rCBV_unidir_, rCBV_bidir_ respectively (32). These maps were normalized by dividing all intensities by the mean intensity of the contralateral NAWM of each rCBV map. “rCBV” subsequently refers to this normalized rCBV. Additionally, the permeability related parameters of K_2_ and K_ep_ for the bidirectional correction algorithm (equation [6]) and K_2_ for unidirectional correction (equation [3]) have been provided.

### Data Analysis and Statistical Method

For each patient the median values of each normalized map (rCBV, rCBV_unidir_, rCBV_bidir_) as well as permeability related parameters (K_2_unidir_, K_2_bidir_ and K_ep_bidir_) within VOIs were computed and used for comparison. These VOIs were CET for enhancing glioma and the NCET for nonenhancing glioma. Data were tested for normality using the Shapiro-Wilk test, with non-parametric statistical tests selected accordingly. Comparison of parameters was done across each glioma group of enhancing and nonenhancing in each dataset by Wilcoxon Signed Rank Test combined with Holm–Bonferroni correction to counteract the problem of multiple comparisons. Moreover, the percentage difference between uncorrected rCBV and both corrected rCBV values were computed and averaged across the enhancing and nonenhancing group per dataset.

### Goodness-of-Fit

Voxelwise goodness of fit was computed *via* calculation of the coefficient of determination, adjusted R-squared for both correction models. This involves the measurement of the difference of $\Delta R_{2}^{*}(t)$ (equation [1]) and its unidirectional (equation [3]) model fit for every time point:

(9) $$R_{adj}^{2}=1-\left[\frac{\left(1-R^{2})(n-1\right)}{n-k-1}\right]$$

(10) $$R^{2}=1-SS_{res}/SS_{tot}$$

where n is the number of time points in the $\Delta R_{2}^{*}(t)$ curve, and k is the number of variables in the model, i.e. 2 and 3 for the unidirectional and bidirectional model, respectively. In equation [10], SS_tot_ is the total sum of squares and SS_res_ is the sum of squares of residuals: 

(11) $$SS_{tot}=\sum_{t}{\left(\Delta R_{2\;}^{*}(t)-\bar{\Delta R_{2}^{*}(t)}\right)}^{2}$$

(12) $$SS_{res}=\sum_{t}{\left(\Delta R_{2\;corr}^{*}(t)-\Delta R_{2}^{*}(t)\right)}^{2}$$

Where $\Delta R_{2}^{*}(t)$ is the uncorrected relaxivity-time curve, $\bar{\Delta R_{2}^{*}(t)}$ is the mean $\Delta R_{2}^{*}(t)$ for each voxel, and $\bar{\Delta R_{2corr}^{*}(t)}$ is the corrected relaxivity-time curve, either bidirectionally or unidirectionally.

The average adjusted R-squared was calculated for the CET and NCET VOIs for each group of patients in each dataset.

### Predicting Glioma Grade With Relative Cerebral Blood Volume

As a proof of principle, we assessed the statistical correlation between rCBV (both corrected and uncorrected) and histopathologic tumor grade using the Spearman rank correlation test (r_s_). In the TCIA dataset all scans were collected shortly before surgery (5 days on average), at which time tumor grade was determined from the resected tumor tissue. This was not the case for the EMC dataset, where scans were acquired at various time points after tumor resection and as a result the initially established tumor grading information might no longer be valid for this imaging dataset to be used for grade prediction.

## Results

**Table 1** lists histopathologic diagnosis of both enhancing and nonenhancing glioma patients in TCIA dataset. The WHO grade II tumors included 13 glioma patients (4 enhancing and 9 nonenhancing); the grade III included 5 glioma patients (1 enhancing and 4 nonenhancing); and the grade IV included 31 glioma patients (30 enhancing and 1 nonenhancing).

**Table 1:** Summary of clinical description of patients in TCIA dataset.

Tumor Grade	Diagnosis	Enhancing Glioma	Nonenhancing Glioma
		Number of patients/Total %	Number of patients/Total %
IV	Glioblastoma multiforme	61.2%	2%
III	Anaplastic Astrocytoma III	2%	6.1%
	Mixed Anaplastic Astrocytoma/Oligodendrogliomas III	0	2%
II	Astrocytoma II	6.1%	6.1%
	Mixed Astrocytoma/Oligodendrogliomas II	0	12.2%
	Ependymoma II	2%	0

**Figure 2:**
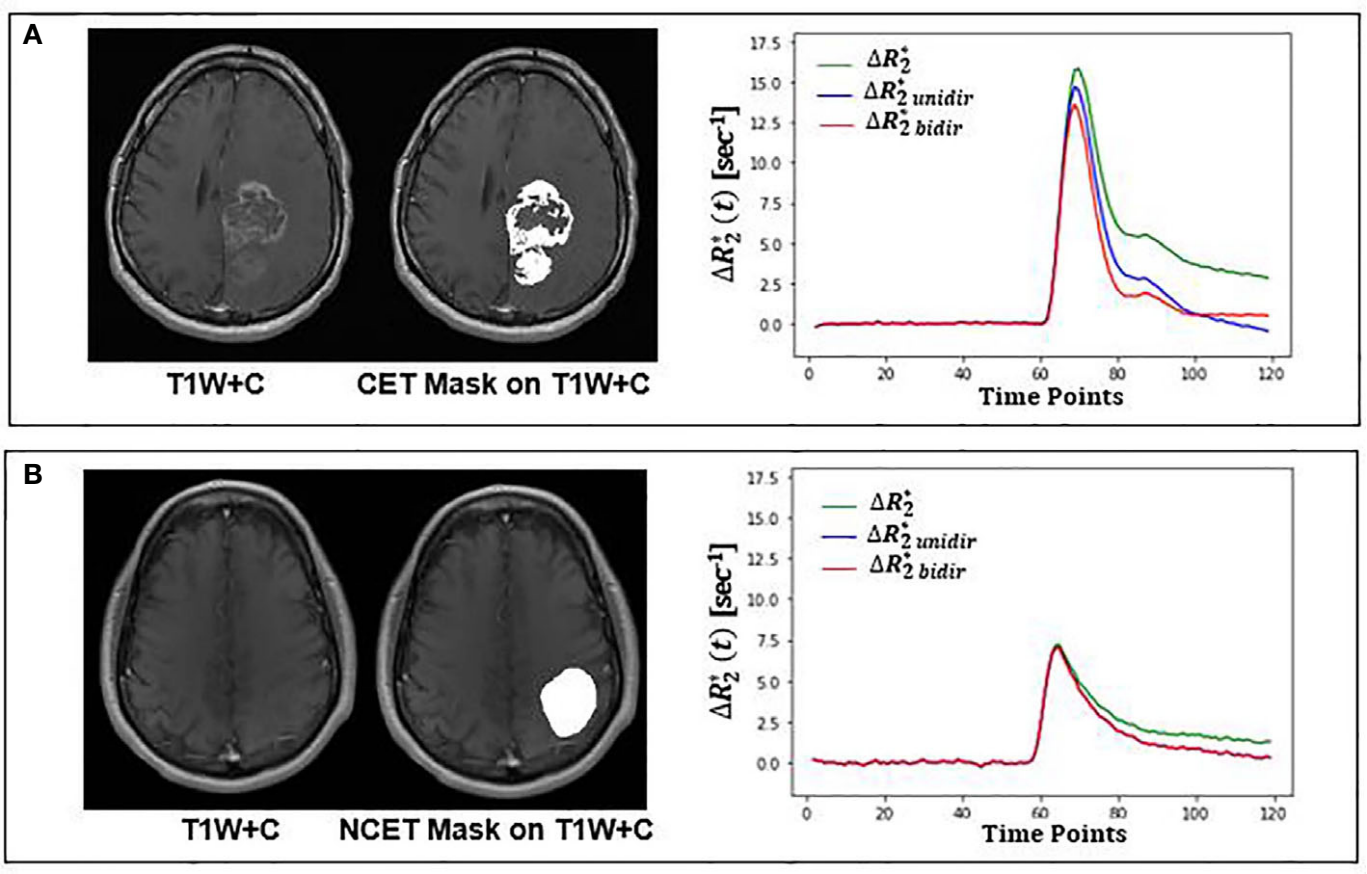
Leakage correction effect on relaxivity-time curve for an enhancing (top) and nonenhancing (bottom) tumor from TCIA dataset. **(A)** Structural T_1_-weighted postcontrast (T1W+C) image overlaid with CET VOI on the left; mean uncorrected (green), bidirectional (red) and unidirectional (blue) corrected relaxivity-time in CET VOI on the right. **(B)** Structural T_1_-weighted postcontrast (T1W+C) image overlaid with NCET VOI on the left; mean uncorrected (green), bidirectional (red) and unidirectional (blue) corrected relaxivity-time in NCET VOI on the right. CET, Contrast Enhanced Tumor, NCET, Non Contrast Enhanced Tumor.

Both unidirectional and bidirectional correction reduced the tail of the uncorrected relaxivity-time curves of the CET VOI in enhancing tumors and the NCET VOI in nonenhancing tumors. Examples of these curves can be seen in **Figure 2**. As exemplified in this figure, a stronger reduction was reached when bidirectional leakage correction was applied. More specifically, the bidirectional corrected relaxivity–time curve in the CET VOI dropped faster initially, but the curve eventually slowed down; however, the unidirectional corrected relaxivity–time curve dropped almost linearly over time. In nonenhancing glioma, the mean relaxivity–time curves of NCET VOI showed smaller differences between either of the two leakage correction algorithms.

**Figure 3:**
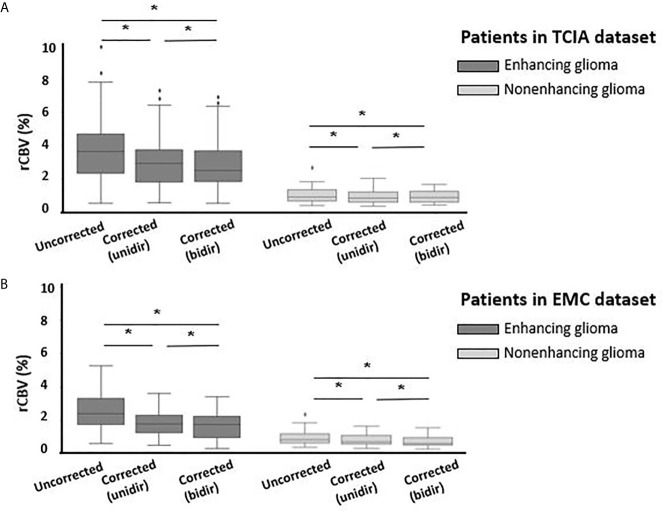
Boxplots of uncorrected, unidirectional corrected and bidirectional corrected rCBV values from left to right **(A)** for enhancing glioma (n = 35) (dark gray) and for nonenhancing glioma (n = 14) (light gray) in TCIA dataset; **(B)** for enhancing glioma (n = 20) (dark gray) and nonenhancing glioma (n = 27) (light gray) in EMC dataset. *Significantly different, p < 0.05.

In the CET VOI (i.e. in enhancing glioma) the mean rCBV was significantly decreased when using either correction algorithm in both datasets. In the TCIA dataset uncorrected rCBV was 4.00 ± 2.11 which significantly (p<0.001) decreased with unidirectional correction to 3.19 ± 1.65 (20.2%) and with bidirectional correction to 2.91 ± 1.55 (27.1%) (**Figure 3**). Similar results were found in the EMC dataset with uncorrected rCBV being 2.5 ± 1.30, decreasing significantly (p<0.001) with unidirectional correction to 1.72 ± 0.84 (31.5%) and with bidirectional correction to 1.59 ± 0.90 (36.6%). In the NCET VOI in nonenhancing glioma, small but significant (p<0.05) differences were observed between uncorrected and corrected rCBV in both datasets when applying either of two leakage correction algorithms (see **Table 2**). Moreover, in both datasets and in both enhancing and nonenhancing tumors bidirectionally corrected rCBV values were significantly lower compared to unidirectionally corrected rCBV.

**Table 2:** Patient averages of uncorrected and corrected rCBV and the resulting P-Value from statistical analysis.

		Uncorrected	Unidirectional Leakage Correction		Bidirectional Leakage Correction	
		rCBV(mean ± std)	rCBV(mean ± std)	Difference percentage(P-Value)	rCBV(mean ± std)	Difference percentage(P-Value)
**TCIA dataset**	**Enhancing Glioma**	4.00 ± 2.11	3.19 ± 1.65	20.2% P < 0.001	2.91 ± 1.55	27.1% P < 0.001
	**Nonenhancing Glioma**	1.42 ± 0.60	1.28 ± 0.46	9.5% P < 0.001	1.24 ± 0.37	12.6% P = 0.02
**EMC dataset**	**Enhancing Glioma**	2.51 ± 1.30	1.72 ± 0.84	31.5% P < 0.001	1.59 ± 0.90	36.6% P < 0.001
	**Nonenhancing Glioma**	0.91 ± 0.46	0.77 ± 0.37	14.6% P < 0.001	0.67 ± 0.34	25.9% P < 0.001

The difference Percentage is the percentage of relative change between each of leakage corrected and uncorrected rCBV.

**Figure 4:**
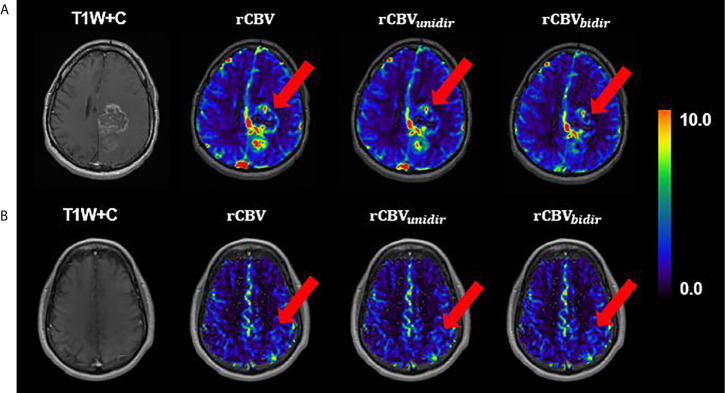
A slice example of T_1_-weighted postcontrast overlaid with uncorrected, unidirectional corrected and bidirectional corrected rCBV maps from left to right, respectively, **(A)** for enhancing glioma patient **(B)** for nonenhancing glioma; both from TCIA dataset.

Visual inspection of uncorrected rCBV and corrected rCBV_unidir_ and rCBV_bidir_ maps is consistent with the above stated findings. As shown in **Figure 4**, the difference between three rCBV maps of a nonenhancing tumor is not clearly perceived, while in the enhancing tumor the difference between rCBV with and without correction is detectable in the CET VOI.

**Figure 5:**
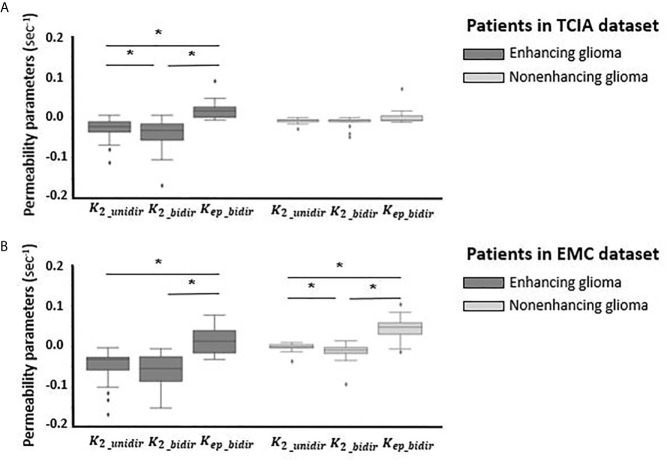
Boxplots of permeability parameters of K_2_ in unidirectional correction method and K_2_ and K_ep_ in bidirectional correction method from left to right **(A)** for enhancing glioma (n = 35) (dark gray) and for nonenhancing glioma (n = 14) (light gray) in TCIA dataset; **(B)** for enhancing glioma (n = 20) (dark gray) and nonenhancing glioma (n = 27) (light gray) in EMC dataset. *Significantly different, p < 0.05.

Variation of permeability parameters of both correction methods has been depicted in **Figure 5** for both datasets. The mean value of K_2_ in both datasets across enhancing and nonenhancing groups is negative, with a close to zero value for nonenhancing ones. This value for enhancing tumors is K_2_unidir_ = -0.03 ± 0.02 (sec^-1^) and K_2_bidir_ = -0.05 ± 0.08 (sec^-1^) for TCIA dataset; K_2_unidir_ = -0.05 ± 0.04 (sec^-1^) and K_2_bidir_ = -0.06 ± 0.04 (sec^-1^) for EMC dataset. The transfer coefficient K_ep_bidir_ that appears in the bidirectional model, representing the extra- to intravascular contrast flux, had a positive mean value of 0.02 ± 0.05 (sec^-1^) and 0.01 ± 0.02 (sec^-1^) for enhancing tumors of EMC and TCIA respectively.

Evaluation of model fitting by averaging adjusted R-squared value across patients showed limited differences between the bidirectional (0.87 ± 0.12) and unidirectional (0.87 ± 0.12) models in TCIA dataset, while in EMC dataset adjusted R-squared of the bidirectional model (0.86 ± 0.05) was slightly higher compared to the unidirectional model (0.83 ± 0.06).

**Figure 6:**
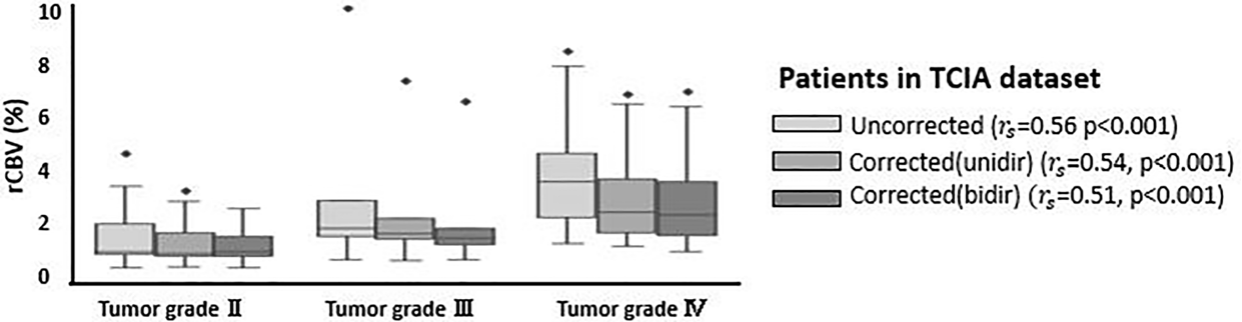
Boxplots of uncorrected, unidirectional corrected and bidirectional corrected rCBV values per each grade (grade II, III, and IV). *r_s_* represents the correlation coefficient between rCBV and tumor grade, derived from Spearman rank correlation test. *Significantly different, p < 0.05.

**Table 3** shows corrected and uncorrected rCBV measurements for each grade and each tumor type in the TCIA dataset. The average rCBV values were higher for grade IV and III compared to grade II and decreased after application of either of leakage correction algorithm. Across the 49 patients in TCIA dataset, tumor grade and rCBV were significantly correlated with or without leakage correction algorithm (see **Figure 6**).

**Table 3:** Patient averages of uncorrected and corrected rCBV for the different tumor types and the grades in TCIA.

Tumor Grade	Diagnosis	Uncorrected		Unidirectional Leakage Correction		Bidirectional Leakage Correction	
		rCBV (mean ± std)		rCBV (mean ± std)		rCBV (mean ± std)	
		Type	grade	Type	grade	Type	grade
IV	**Glioblastoma multiforme**	3.90 ± 1.87	3.90 ± 1.87	3.13 ± 1.53	3.13 ± 1.53	2.88 ± 1.46	2.88 ± 1.46
III	**Anaplastic Astrocytoma** III	3.57 ± 4.25	3.45 ± 3.69	2.83 ± 2.99	2.73 ± 2.6	2.56 ± 2.66	2.44 ± 2.32
	**Mixed Anaplastic Astrocytoma/Oligodendrogliomas** III	2.94		2.31		1.94	
II	**Astrocytoma** II	1.58 ± 1.05	1.66 ± 1.18	1.46 ± 0.86	1.45 ± 0.84	1.44 ± 0.79	1.38 ± 0.67
	**Mixed Astrocytoma/Oligodendrogliomas** II	1.24 ± 0.48		1.13 ± 0.4		1.14 ± 0.38	
	**Ependymoma** II	4.65		3.29		2.48	

## Discussion

This study investigated the effect of two known leakage correction algorithms on rCBV measurements in both enhancing and nonenhancing glioma in two independent datasets. The leakage correction algorithms used in this study are based on unidirectional contrast agent transport from the intravascular to extravascular spaces and on bidirectional contrast agent transport between these two spaces. The result of this study showed that in enhancing glioma, when the BBB is disrupted, application of either of these two leakage correction methods decreased rCBV measurements. The decrease in rCBV measurements in enhancing glioma after applying leakage correction algorithms likely originates from initial rCBV overestimation due to dominance of ${\text{T}}_{2}^{*}$ effects in the leaky area.

We have seen different effect size of leakage correction in the two investigated datasets, with a stronger reduction on average in rCBV for the EMC dataset. Previously it has been shown that the leakage of contrast agent into the extravascular extracellular space results in increased T_1_ and ${\text{T}}_{2}^{*}$ effect, by shortening both T_1_ and ${\text{T}}_{2}^{*}$ relaxation time. Depending on which of two has dominant effect in the leaky area, the tail of relaxivity-time curve gets artificially either lower or upper than the baseline (11). In the EMC protocol applying higher dose of preload, combined with long T_E_ and high FA, the measured signal would be less sensitive to change in T_1_ effect and more sensitive to change in ${\text{T}}_{2}^{*}$ effect, compared to TCIA dataset. Stronger ${\text{T}}_{2}^{*}$ effect dominance in the enhanced area results in highly elevated tail in relaxivity-time curve. Thus, when applying either of leakage correction algorithms on the curve, the effect size would appear stronger.

In line with previous consensus (20) our result highlights the necessity of using either leakage correction algorithm for rCBV measurements in enhancing glioma. However, when using these algorithms in the absence of contrast agent leakage effects, interpretation should be done with caution. The result of this study shows that both leakage correction algorithms significantly altered rCBV estimation in nonenhancing glioma. Although, this alteration was not noticeable in most patients, there might be a risk of overfitting in using these leakage correction algorithms in nonenhancing glioma. One explanation for this phenomenon could be the elevated steady-state contrast agent concentration in the vasculature after first passage. This might interfere with the performance of these algorithms and cause rCBV misestimation after application of leakage correction methods. Another possible explanation for this result could be that these algorithms are able to detect subtle leakage effects which are not yet clearly visible on a T_1_ postcontrast image. Since there are no histopathological rCBV measurements to serve as a gold standard for rCBV in nonenhancing tumor caution needs to be taken with application of leakage correction in nonenhancing areas.

Comparing the performance of the uni- vs bidirectional leakage algorithms, each method has its own pros and cons. The goodness-of-fit analysis showed slightly higher adjusted R-squared for bidirectional method. However, the bidirectional leakage correction takes twice as long to compute as the unidirectional one, which may be clinically undesirable. Analyses of the permeability parameters for nonenhancing patients shows that K_2_ obtained from unidirectional algorithm is plausibly close to zero in both datasets, whereas K_2_ and K_ep_ resulted from bidirectional model are changing in a broader range specially for nonenhancing group in EMC datasets. The likely reason for this unexpected behavior could be the number of time points (50 time points) collected in EMC dataset, as previous investigations have indicated that the leakage correction algorithm performed best with the collection of 120 time points (14).

It is worthwhile to note that in TCIA dataset, including both enhancing and nonenhancing glioma, a significant correlation was found between tumor grading and all three rCBV calculations, including uncorrected, unidirectionally and bidirectionally corrected rCBV. Therefore, the unexpected finding of significant effects of leakage correction on rCBV on nonenhancing tumor may not be an issue for standard application of leakage correction in clinical settings. However, generalization of this finding requires further corroboration in multiple clinical studies.

A limitation of this study is the retrospective nature which leads to both datasets not following the standardized DSC-MRI acquisition protocol (20). In consensus study it has been suggested that using a full-dose of 0.1 mmol/kg for both preload and bolus injection dose, along with DSC acquisition parameters of 60° for FA and T_E_ of 40–50 ms at 1.5 T and 20–35 ms at 3 T, provide overall best accuracy and precision for rCBV estimates. As with many retrospective studies, the DSC-MRI acquisition protocol used for both TCIA and EMC datasets do not fall within the standardized acquisition protocol, as described in the method section. For instance, in TCIA dataset the preload is 0.05 mmol/kg which is half of what is recommended currently; and in EMC dataset, the dose protocol is not based on weight but on contrast volume (7.5ml). With the standard “full dose” defined as 0.1 mmol/kg, only patients weighing 75 kg (~165 lb) received a full dose, while patients under and over this weight would receive more than and less than a full dose, respectively. Therefore, future work should be focused on examining datasets with the most recent standard protocol and a ground truth for MRI-derived perfusion parameter utilizing spatially-correlated biopsy samples.

In summary, this work evaluated the effect of leakage correction on rCBV estimates, indicating stronger effects for bidirectional than for unidirectional leakage correction as well as larger effects in enhancing tumors than in nonenhancing tumors. From a clinical perspective, our work highlights that using rCBV as a universal biomarker still requires further development in standardization of validation of both acquisition and post-processing procedures. The fact that the application of a correction algorithm affects the estimated rCBV indicates that the use of published threshold values (8) for determining tumor type, molecular profile or grade has to be done with great caution, taking the methodology for establishing such thresholds into account.

## Data Availability Statement

A publicly available datasets was analyzed in this study. Data can be found here: https://wiki.cancerimagingarchive.net/display/Public/QIN-BRAIN-DSC-MRI.

## Ethics Statement

The studies involving human participants were reviewed and approved by the first dataset used in this study. “Glioma DSC-MRI Perfusion Data” is publicly available in The Cancer Imaging Archive (TCIA). For the second dataset, the complete protocol is part of routine clinical imaging, and all patients provided informed written consent to have their information stored in an Institutional Review Board Approved Neuro-Oncology database for use in future investigations. The patients/participants provided their written informed consent to participate in this study.

## Author Contributions

The study was conceptualized by EW, PC, and MC. The manuscript was written by FA. Image processing and statistical analysis were done by FA with the help of KG for tumor segmentation and PC for statistical analysis. The result has been evaluated by FA, EW, PC, and MC for the technical point of view and MS for the clinical point of view as an experienced radiologist. MS also provided one of the clinical datasets used in this work. All authors helped in interpreting the data and critically reviewed the manuscript and commented on the final version. All authors contributed to the article and approved the submitted version.

## Funding

FA conducted this work funded by a Short Term Scientific Mission from Glioma MR Imaging 2.0, an Action funded by the European Cooperation in Science and Technology. EW is funded by a “Veni Vernieuwingsimpuls” from the Dutch Research Council entitled “Food for thought: Oxygen delivery to the brain,” grant number 91619121.

## Conflict of Interest

The authors declare that the research was conducted in the absence of any commercial or financial relationships that could be construed as a potential conflict of interest.
